# Analysis of the safety and efficacy of laparoscopic gastrojejunostomy following neoadjuvant chemotherapy for gastric pyloric obstruction

**DOI:** 10.3389/fonc.2025.1430761

**Published:** 2025-03-18

**Authors:** Bo Hu, Yishan Zeng

**Affiliations:** Department of Gastrointestinal Surgery, Xiamen Humanity Hospital, Xiamen, China

**Keywords:** gastrojejunostomy, neoadjuvant chemotherapy, gastric pyloric obstruction, endoscopic stent, gastric cancer

## Abstract

**Objective:**

To explore the safety and feasibility of laparoscopic gastrojejunostomy combined with neoadjuvant chemotherapy (NACT) in patients with locally advanced gastric cancer and pyloric obstruction.

**Methods:**

We included patients with locally advanced gastric cancer who underwent laparoscopic gastrojejunostomy (LGJ) or endoscopic stenting (ES) between May 2017 and October 2022. The prognostic nutritional index (PNI) was used to evaluate the patient nutritional status. Platelet-to-lymphocyte ratio (PLR) and neutrophil-to-lymphocyte ratios were used to evaluate the inflammatory status of patients. The Kaplan–Meier method was used to analyze survival conditions, and the log-rank test was used to compare survival differences. A multivariate logistic regression analysis was performed to identify the factors related that might affect the prognosis.

**Results:**

During the study period, 41 patients received LGJ and 37 patients received endoscopic stenting (ES). Patients in the ES group had higher rates of postoperative complications, particularly bleeding (0 vs. 16.2%, P<0.05). After two cycles NACT, the proportion of PNI≥45 patients in LGJ group was significantly higher than that in ES group (P<0.05). Furthermore, the proportion of patients with PLR<162 in the ES group was significantly higher than that in the LGJ group (P<0.05), and compared to the ES group, patients in the LGJ group were able to tolerate more cycles of NACT (6 vs. 4 cycles). A higher median survival time was observed in the LGJ group, and the multivariate logistic regression analysis confirmed treatment selection as an independent risk factor for overall survival (HR, 6.362; 95% CI:3.285–12.321, P<0.001).

**Conclusion:**

NACT after LGJ shows potential for reducing tumor stage and improving patient prognosis.

## 1 Introduction

Gastric cancer is one of the most common malignancies worldwide and has a high mortality rate (1). Due to the lack of clinical symptom specificity, most clinically diagnosed gastric cancers are at advanced stages, and the prognosis is poor at such stages due to the possibility of metastasis (2). However, with the continuous improvements in medical treatment, neoadjuvant chemotherapy (NACT) before surgery has gradually become the first choice for the clinical treatment of patients with advanced gastric cancer (3). Notably, the MAGIC trial showed that perioperative chemotherapy extended the survival of patients with gastric cancer by 5 years (23–36%), heralding a new era of neoadjuvant therapy (4).

Preoperative NACT is designed to improve the prognosis of patients by reducing tumor stage, improving the radical degree of surgery, and removing tumor cell micrometastases; the treatment completion rate of adjuvant chemotherapy among patients is higher preoperatively than postoperatively. Pyloric obstruction is a common complication of distal gastric cancer (5, 6), and the symptoms of vomiting and difficulty eating seriously hinder the development of enteral nutrition support. This results in poor nutritional and metabolic status in such patients, which makes it difficult for the patients to accept neoadjuvant therapy (5). To ameliorate this problem, relieving the symptoms of pyloric obstruction is important and endoscopic stenting (ES) and gastrojejunostomy are two commonly used palliative treatments for pyloric obstruction (5). In clinical practice, ES is usually recommended for older patients with poor physical activity and difficulty in tolerating surgery to avoid the trauma caused by surgery (7). However, the limited patency time of the gastric outlet and high recurrence rate of stent implantation limit the development of enteral nutrition and the application of oral chemotherapy (7, 8).

In recent years, with the development of laparoscopic technology and the accumulation of clinical trial evidence, the application scope of laparoscopic surgery has gradually expanded from early to locally advanced gastric cancer. Compared with traditional open surgery, laparoscopic surgery has the advantages of less postoperative pain and faster recovery (9–12). However, the efficacy of laparoscopic gastrojejunostomy (LGJ) combined with NACT in patients with locally advanced gastric cancer complicated by pyloric obstruction has not been fully clarified. Notably, few studies have reported on the use of LGJ combined with neoadjuvant therapy in patients with gastric cancer complicated by pyloric obstruction. Therefore, the purpose of this study was to review our clinical experience with the treatment of patients with locally advanced gastric cancer complicated by pyloric obstruction and to evaluate the feasibility of neoadjuvant therapy after LGJ.

## 2 Materials and methods

### 2.1 Patients

Patients with locally advanced gastric cancer who underwent LGJ or ES in the Department of Gastrointestinal Surgery of Xiamen Humanities Hospital and Xiamen Hospital of T.C.M., between May 2017 and October 2022 were included. The inclusion criteria were as follows: (1) gastric adenocarcinoma confirmed by gastroscopic biopsy and pathological examination and (2) in accordance with the AJCC and UICC eights edition gastric cancer staging systems (13) endoscopic examination performed with computed tomography (CT) to diagnose pyloric obstruction (3); in the Eastern United States, tumors within the physical status score (Eastern Cooperative Oncology Group Performance status, ECOG PS) (14) of 0 or 1; and (4) complete clinical data. The exclusion criteria were as follows: (1) gastrointestinal obstruction other than gastric pyloric obstruction; (2) gastric adenocarcinoma combined with other malignant tumors; (3) receiving other antitumor therapy such as radiotherapy or chemotherapy before surgery; and (4) severe insufficiency of important organs such as the heart, lungs, and kidneys. According to the above inclusion and exclusion criteria, a total of 78 patients with pyloric obstruction were enrolled in our study. Written informed consent was obtained from all the patients, and this study was approved by the Ethics Committee of Xiamen Humanity Hospital. All study procedures were performed in accordance with the Declaration of Helsinki of 1964 and its later version.

### 2.2 Treatments

The multidisciplinary treatment team explained the advantages and potential risks of NACT to each patient and selected treatment strategies according to the general condition, disease characteristics, and economic condition of each patient. Enteral nutrition and early parenteral nutrition support were initiated after LGJ or ES, and all patients received additional enteral nutrition support during hospitalization. Chemotherapy was initiated 7–14 d after LGJ or ES treatment. Chemotherapy regimen EOX regimen: epirubicin 100 mg/m^2^ D1, oxaliplatin 130 mg/m^2^ D1, capecitabine 825 mg/m^2^ D1-14, 3 weeks. Patients who receive neoadjuvant therapy typically receive 2–8 cycles of preoperative chemotherapy. If deterioration of the disease or intolerable side effects of chemotherapy occurred, patients could refuse to continue the treatment. CT was performed every two cycles after chemotherapy, and radical surgical treatment was performed after a multidisciplinary discussion, to evaluate radical resection of the tumor. Tumors that responded to the treatment were evaluated as either attaining a complete response (CR) or a partial response (PR) (15).

### 2.3 Clinical and pathological outcomes

The following baseline data of patients were collected and analyzed: age, sex, body mass index (BMI), ECOG PS, Borrmann classification, Lauren classification, and cT stage.

For the nutritional status assessment, the prognosis nutrition index (PNI) (16), Nutritional Risk Screening 2002 (NRS-2002) (17), PG-SGA assessments (18) and Spitzer QOL-Index (19) were used. Inflammatory status assessments included platelet-to-lymphocyte ratio (PLR) and neutrophil-to-lymphocyte ratio (NLR), and the patients were divided into groups based on PLR (<162 vs. ≥162) and NLR (<2.5 vs. ≥2.5) (16, 20–23). Gastric outlet obstruction (GOOSS) was used to assess oral difficulty as follows: 0, unable to eat, 1 liquid, 2 half liquid; and 3, normal diet (24). Nutritional status, inflammatory status, and eating difficulties were evaluated before LGJ or ES and after the two cycles of chemotherapy.

The postoperative recovery evaluation included postoperative complications, time to first aerofluxus, and length of hospitalization. Postoperative complications were defined as those occurring within 30 d of surgery.

Chemotherapy efficacy was evaluated based on the Response Evaluation Criteria in Solid Tumors guidelines (version 1.0). CR, PR, stable disease, progressive disease (25). Toxicity was assessed according to the Common Toxicity Criteria for Adverse Events (version 3.0) (26).

The postoperative pathology included pT, pN, and pTNM stages; tumor regression grade (5); and pathological CR. The postoperative specimens were evaluated by more than two pathologists.

For follow-up, patients were followed up through outpatient and telephone visits after discharge, and the follow-up period lasted until September 2023. Routine abdominal CT was performed every 3 months for 2 years, every 6 months for 2–5 years, and annually for 5 years. OS (overall survival) is defined as the time from the initiation of treatment to death. DFS (disease-free survival) is defined as the time from the initiation of treatment to disease recurrence or death. PFS (progression-free survival) is defined as the time from the initiation of treatment to disease progression or death.

### 2.4 Statistical analysis

SPSS24.0 statistical software was used for statistical analysis. The Pearson’s chi-squared test, Fisher’s exact probability method, and t-test were used for comparisons among all groups. The Kaplan–Meier method was used to draw a survival curve and calculate the cumulative survival rate. Cox regression was performed to find independent factors affecting OS, DFS and PFS after treatments. Hazard ratios (HR) and 95% confidence intervals (95% CI) were calculated. The difference in OS, DFS and PFS between the two groups were compared using the log-rank test, and differences with P<0.05 were considered statistically significant.

## 3 Results

### 3.1 Baseline characteristics

No statistically significant differences in age, sex, BMI, ECOG PS, GOOSS, NRS-2002, PG-SGA category, PNI, NLR, PLR, overall QOL, Borrmann score, or cT stage were observed between the two patient cohorts (all P>0.05) (**Table 1**).

**Table 1:** Patient baseline characteristics in the LGJ and ES groups.

	LGJ (n=41)	ES (n=37)	*P*
Age (y)	59.5±6.5	61.5±7.5	0.216
Sex			0.297
Male	32 (78.0)	25 (67.6)	
Female	9 (22.0)	12 (32.4)	
BMI (kg/m^2^)	23.2±1.8	22.7±2.8	0.392
ECOG PS			0.891
0	25 (61.0)	22 (59.5)	
1	16 (39.0)	15 (40.5)	
GOOSS (0/1)			0.112
0	17 (41.5)	22 (59.5)	
1	24 (58.5)	15 (40.5)	
NRS 2002 scale			0.916
<3	2 (4.9)	2 (5.4)	
≥3	39 (95.1)	35 (94.6)	
PG-SGA category			0.731
B	3 (7.3)	2 (5.4)	
C	38 (92.7)	35 (94.6)	
PNI			0.804
<45	32 (78.0)	28 (75.7)	
≥45	9 (22.0)	9 (24.3)	
NLR			0.964
<2.5	12 (29.3)	11 (29.7)	
≥2.5	29 (70.7)	26 (70.3)	
PLR			0.297
<162	9 (22.0)	12 (32.4)	
≥162	32 (78.0)	25 (67.6)	
Overall QOL	6 (4–9)	6 (4–10)	0.983
Borrmann type			0.223
I	1 (2.4)	3 (8.1)	
II	8 (19.5)	4 (10.8)	
III	31 (75.6)	26 (70.3)	
IV	1 (2.4)	4 (10.8)	
cT stage			0.552
T3	5 (12.2)	3 (8.1)	
T4	36 (87.8)	34 (91.9)	

PS, Performance status; GOOSS, Gastric outlet obstruction scoring system; NRS 2002, Nutrition Risk Screening 2002; PG-SGA, Patient-Generated Subjective Global Assessment; PNI, Prognostic Nutritional Index; NLR, Neutrophil to lymphocyte ratio; PLR, Platelet to lymphocyte ratio; QOL Quality of life.

### 3.2 Clinical and pathological outcomes

Patients in the LGJ group had higher rates of postoperative complications, particularly bleeding (P<0.05) and shorter aerofluxus time and hospital stay after treatment (P<0.05). All complications resolved after conservative treatment.

After LGJ or ES, 95.1% of the patients in the LGJ group and 86.5% of the patients in the ES group returned to their normal diets. After two cycles NACT, the proportions of patients with PNI≥45, NRS 2002 scale <3 and well-nourished (PG-SGA category A) in the LGJ group was significantly higher than that in the ES group (P<0.05). And the overall QOL was also significantly higher in the LGJ group (P<0.05). Furthermore, the proportion of patients with PLR<162 in the ES group was significantly higher than that in the LGJ group (P<0.05). Compared with the ES group, patients in the LGJ group were also able to tolerate more cycles of NACT (6 vs. 4 cycles), presented a significantly higher objective response rate (80.5% vs. 18.9%), fewer adverse events especially neutropenia (12.2% vs. 35.1%) and thrombocytopenia (14.6% vs. 40.5%), and underwent a greater number of radical surgeries (all P<0.05) (**Table 2**).

**Table 2:** Comparison of clinical outcome between the LGJ and ES groups.

	LGJ (n=41)	ES (n=37)	*P*
Postoperative complications			
Bleeding	0	6 (16.2)	0.007
Reobstruction	0	3 (8.1)	0.063
Perforation	1 (2.4)	3 (8.1)	0.257
The first aerofluxus time (days)	3 (1-5)	1 (1-4)	<0.001
Hospital stay after treatment (days)	3 (2-6)	2 (2-6)	<0.001
GOOSS 3 achieved	39 (95.1)	32 (86.5)	0.183
NACT cycles	6 (2-8)	4 (2-6)	<0.001
Response			<0.001
CR	4 (9.8)	1 (2.7)	
PR	29 (70.7)	6 (16.2)	
SD	5 (12.2)	21 (56.8)	
PD	3 (7.3)	9 (24.3)	
NRS 2002 scale			0.043
<3	15 (36.6)	6 (16.2)	
≥3	26 (63.4)	31 (83.8)	
PG-SGA category			<0.001
A	8 (19.5)	0	
B	33 (80.5)	7 (18.9)	
C	0	30 (81.1)	
PNI			<0.001
<45	19 (46.3)	36 (97.3)	
≥45	22 (53.7)	1 (2.7)	
NLR			0.964
<2.5	12 (29.3)	11 (29.7)	
≥2.5	29 (70.7)	26 (70.3)	
PLR			0.004
<162	22 (53.7)	8 (21.6)	
≥162	19 (46.3)	29 (78.4)	
Overall QOL	8 (4-10)	6 (4-10)	<0.001
Adverse events (grade 3/4)			
Anemia	5 (12.2)	4 (10.8)	0.848
Neutropenia	5 (12.2)	12 (35.1)	0.031
Thrombocytopenia	6 (14.6)	15 (40.5)	0.010
Elevate ALT	4 (9.8)	5 (13.5)	0.604
Elevate AST	4 (9.8)	5 (13.5)	0.604
Diarrhea	2 (4.9)	1 (2.7)	0.618
Radical surgery	33 (80.5)	7 (18.9)	<0.001

GOOSS, Gastric outlet obstruction scoring system; NACT, neoadjuvant chemotherapy; NRS 2002, Nutrition Risk Screening 2002; PG-SGA, Patient-Generated Subjective Global Assessment; PNI, Prognostic Nutritional Index; NLR, Neutrophil to lymphocyte ratio; PLR, Platelet to lymphocyte ratio;QOL; Quality of life; ALT, Alanine Aminotransferase; AST, Aspartate Aminotransferase.

After a radical surgery, a lower pTNM stage was observed in the LGJ group (P<0.05), and five (15.2%) pathological CRs were reached (**Table 3**).

**Table 3:** Comparison of pathologies between the LGJ and ES groups.

	LGJ (n=33)	ES (n=7)	P
pT stage			0.052
0-2	29 (87.9)	4 (57.1)	
3-4	4 (12.1)	3 (42.9)	
pN stage			0.747
0	21 (63.6)	4 (57.1)	
1-3	12 (36.4)	3 (42.9)	
pTNM stage			0.020
0-II	32 (97.0)	5 (71.4)	
III	1 (3.0)	2 (28.6)	
Tumor regression grades			0.550
0	5 (15.2)	0	
1	12 (36.4)	4 (57.1)	
2	14 (42.4)	3 (42.9)	
3	2 (6.1)	0	
Pathological complete response	5 (15.2)	0	0.271

### 3.3 Survival analysis

**Figure 1:**
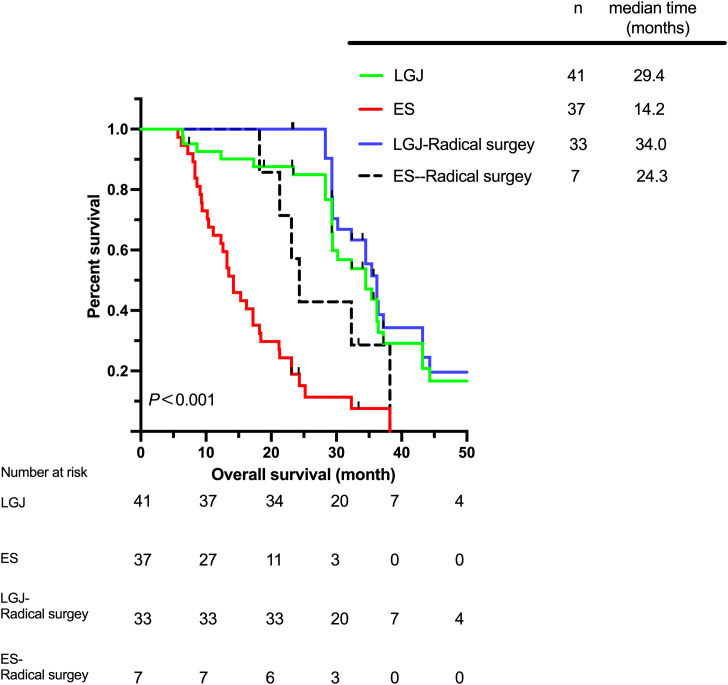
Overall survival according to different treatments.

**Figure 2:**
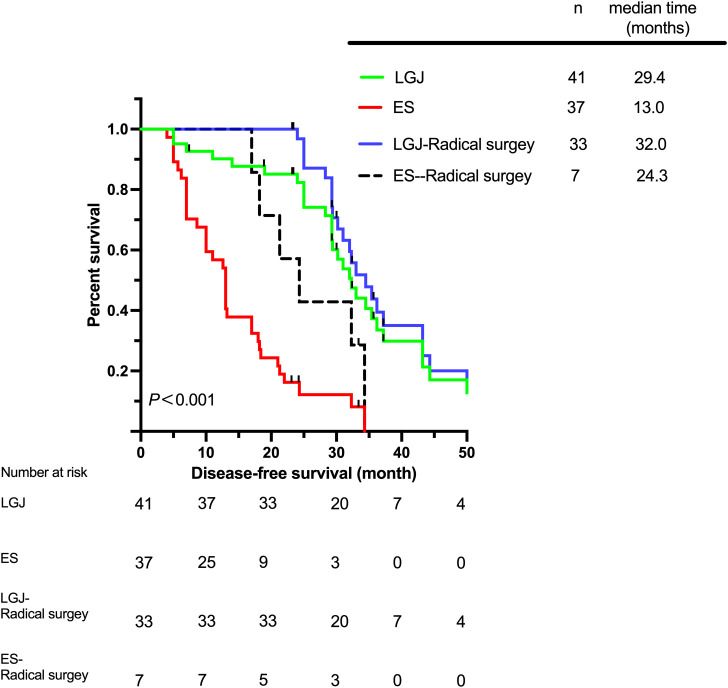
Disease-free survival according to different treatments.

**Figure 3:**
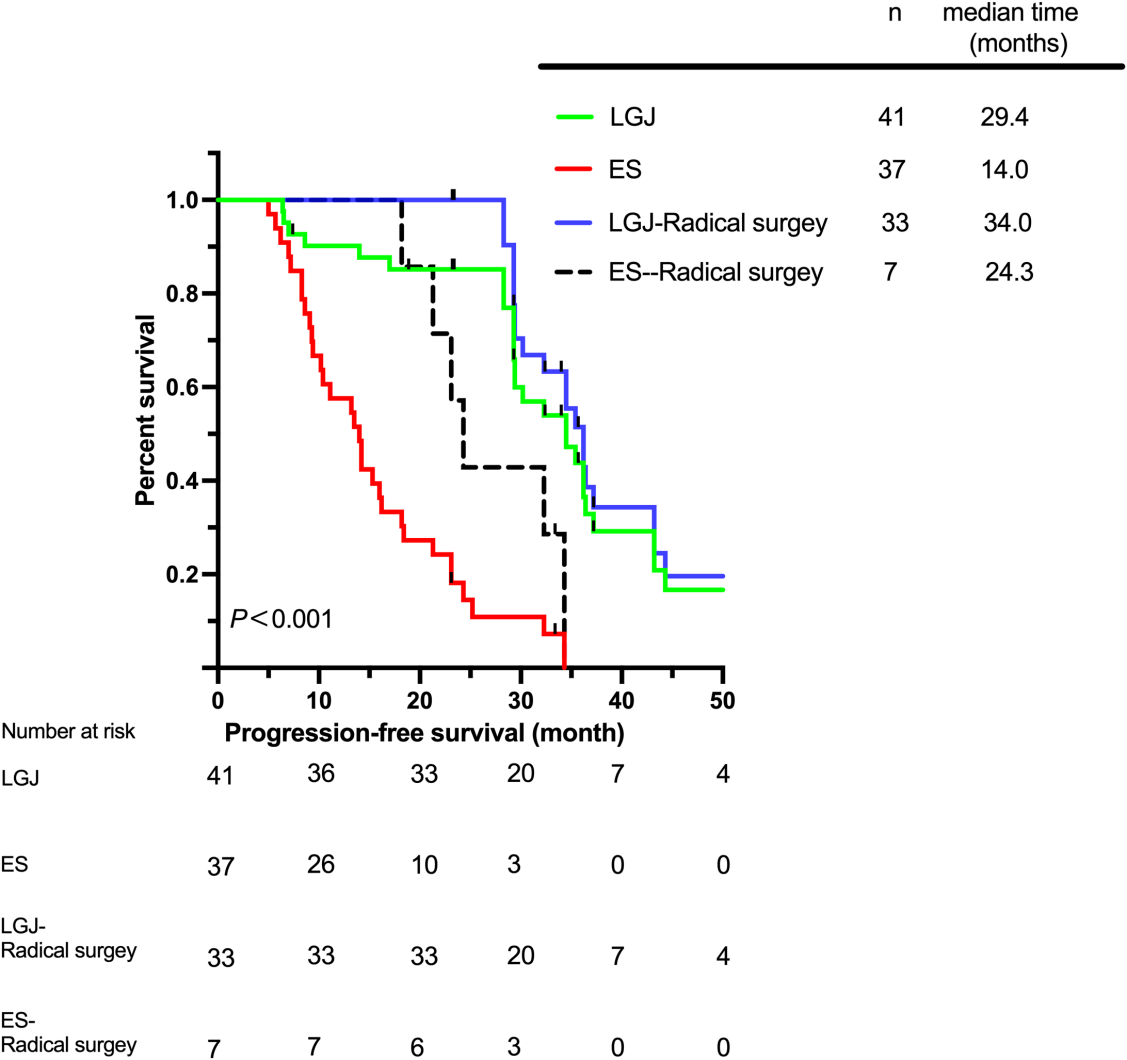
Progression-free survival according to different treatments.

The total median survival time (MST) of the two groups was 23.3 (5.7–64.3) months, while the MST of the LGJ group was 29.4 (6.4–64.3) months, and the MST of the ES group was 14.2 (5.7–38.2) months. The 3-year OS rates of patients in the LGJ and ES groups were 29.3% and 2.7%, respectively. The highest MST was observed in the LGJ group for patients who underwent radical surgery, for whom the MST was 34.0 (23.2–64.3) months (**Figure 1**). We have also observed similar conclusions in terms of DFS and PFS (**Figures 2**, **3**). The multivariate logistic regression analysis confirmed that treatment selection was an independent risk factor for OS (hazard ratio, 6.286; 95% confidence interval: 3.322–11.894, P<0.001) (**Table 4**), DFS (hazard ratio, 5.335; 95% confidence interval: 2.851–9.982, P<0.001) (**Table 5**) and PFS (hazard ratio, 5.862; 95% confidence interval: 2.982–11.521, P<0.001) (**Table 6**).

**Table 4:** Univariate and multivariate analyses for OS.

Variable	Hazard ratio	95% CI	*P* value
Univariate analysis			
Age (≥65/<65)	0.707	0.406-1.229	0.219
Sex (male/female)	1.222	0.690-2.162	0.492
NRS 2002 scale (<3/≥3)	0.709	0.172-2.919	0.634
PG-SGA category (B/C)	0.956	0.346-2.643	0.930
PNI (≥45/<45)	0.806	0.443-1.469	0.482
PLR (≥162/<162)	1.166	0.659-2.065	0.598
NLR (≥2.5/<2.5)	0.833	0.491-1.411	0.497
Overall QOL (improved or stable/ decreased)	0.423	0.179-0.999	0.050
Treatment selection (LGJ/ES)	6.165	3.293-11.542	<0.001
Multivariate analysis			
Age (≥65/<65)	1.111	0.613-2.015	0.728
PNI (≥45/<45)	1.259	0.688-2.304	0.455
PLR (≥162/<162)	0.675	0.352-1.296	0.238
NLR (≥2.5/<2.5)	1.247	0.711-2.189	0.441
Overall QOL (improved or stable/ decreased)	1.018	0.369-2.805	0.973
Treatment selection (LGJ/ES)	6.362	3.285-12.321	<0.001

OS, Overall survival; NRS 2002, Nutrition Risk Screening 2002; PG-SGA, Patient-Generated Subjective Global Assessment; PNI, Prognostic Nutritional Index; PLR, Platelet to lymphocyte ratio; NLR, Neutrophil to lymphocyte ratio; QOL, Quality of life.

**Table 5:** Univariate and multivariate analyses for DFS.

Variable	Hazard ratio	95% CI	*P* value
Univariate analysis			
Age (≥65/<65)	1.373	0.791-2.381	0.260
Sex (male/female)	0.826	0.467-1.461	0.512
NRS 2002 scale(<3/≥3)	0.672	0.163-2.763	0.582
PG-SGA category(B/C)	1.020	0.368-2.831	0.969
PNI (≥45/<45)	1.192	0.654-2.171	0.566
PLR (≥162/<162)	0.897	0.507-1.587	0.708
NLR (≥2.5/<2.5)	1.189	0.701-2.015	0.521
Overall QOL(improved or stable/ decreased)	0.465	0.198-1.093	0.079
Treatment selection (LGJ/ES)	5.109	2.827-9.233	<0.001
Multivariate analysis			
Age (≥65/<65)	1.149	0.635-2.077	0.646
PNI (≥45/<45)	1.174	0.639-2.156	0.605
PLR (≥162/<162)	0.746	0.393-1.419	0.372
NLR (≥2.5/<2.5)	1.212	0.694-2.116	0.500
Overall QOL (improved or stable/ decreased)	1.159	0.423-3.174	0.774
Treatment selection (LGJ/ES)	5.335	2.851-9.982	<0.001

DFS, Disease-free survival; NRS 2002, Nutrition Risk Screening 2002; PG-SGA, Patient-Generated Subjective Global Assessment; PNI, Prognostic Nutritional Index; PLR, Platelet to lymphocyte ratio; NLR, Neutrophil to lymphocyte ratio; QOL, Quality of life.

**Table 6:** Univariate and multivariate analyses for PFS.

Variable	Hazard ratio	95% CI	*P* value
Univariate analysis			
Age (≥65/<65)	1.416	0.802-2.500	0.230
Sex (male/female)	0.922	0.505-1.683	0.792
NRS 2002 scale(<3/≥3)	0.750	0.182-3.093	0.691
PG-SGA category(B/C)	0.992	0.358-2.749	0.988
PNI (≥45/<45)	1.319	0.710-2.450	0.381
PLR (≥162/<162)	0.822	0.456-1.481	0.514
NLR (≥2.5/<2.5)	1.199	0.698-2.059	0.510
Overall QOL (improved or stable/ decreased)	0.515	0.203-1.310	0.164
Treatment selection (LGJ/ES)	5.499	2.898-10.433	<0.001
Multivariate analysis			
Age (≥65/<65)	1.157	0.631-2.119	0.637
PNI (≥45/<45)	1.359	0.728-2.537	0.335
PLR (≥162/<162)	0.684	0.353-1.323	0.259
NLR (≥2.5/<2.5)	1.252	0.708-2.214	0.439
Overall QOL (improved or stable/ decreased)	1.224	0.413-3.629	0.716
Treatment selection (LGJ/ES)	5.862	2.982-11.521	<0.001

PFS, Progression-free survival; NRS 2002, Nutrition Risk Screening 2002; PG-SGA, Patient-Generated Subjective Global Assessment; PNI, Prognostic Nutritional Index; PLR, Platelet to lymphocyte ratio; NLR, Neutrophil to lymphocyte ratio; QOL, Quality of life.

## 4 Discussion

Difficulties remain in the treatment of advanced gastric cancer, such as a low radical resection rate, high recurrence rate, and high mortality. Surgical resection combined with lymph node dissection is the main mode of treatment for advanced gastric cancer; however, ideal results are rarely achieved with surgical treatment alone. Comprehensive perioperative treatment based on surgery, combined chemotherapy, and radiotherapy has become the global consensus (27). Pyloric obstruction is a common complication of advanced distal gastric cancer, and the symptoms of vomiting, abdominal distension, and difficulty in eating caused by pyloric obstruction seriously hinder the development of enteral nutrition support and result in extremely poor nutritional and metabolic status and reduce the ability of patients to accept NACT (14). Palliative resection is often used for patients with advanced gastric cancer and obstructive symptoms; however, it has little impact on their long-term prognosis (28). Some studies (29) have shown that after palliative resection, tumor cells are released into the blood, and the activity of the remaining tumor cells is enhanced under surgical stress and inflammatory response, which greatly enhances tumor proliferation ability and invasion and metastasis. This study compared the long-term prognostic effects of different pyloric obstruction disconnection methods combined with NACT and evaluated the effects of different treatment modes on OS. These findings confirm that patients receiving LGJ have better nutritional statuses and better responses to chemotherapy, this providing a new clinical treatment option.

This study found that ES had a shorter postoperative exhaust time and hospital stay than LGJ; however, it also had a higher incidence of postoperative complications, especially postoperative bleeding (0 vs. 16.2%, P=0.007). Moreover, patients with LGJ had a better nutritional inflammatory state and received NACT for a longer period. Previous studies have suggested that stent implantation is limited and have shown that food and tumor blockage by the stent will lead to recurrence of the obstruction and that stent relocation also requires follow-up treatment. In some patients, stent perforation and other complications may occur that can interfere with treatment. LGJ reduces the stress response and avoids problems such as endoscopic catheterization failure due to obstruction and stent displacement caused by tumor progression (30).

Radical surgery plays a crucial role in the long-term prognosis of patients with advanced gastric cancer (31, 32). Yoshio et al. (5) conducted a multi-center cohort study and found that when stent implantation and gastrojejunostomy were performed, only 1% and 15% of patients with combined pyloric obstruction, respectively, underwent radical resection. One possible reason is that pyloric obstruction causes chronic malnutrition and reduces the ability of patients with gastric cancer to receive follow-up treatment. Notably, LGJ can bypass the obstruction site without stimulating the primary tumor, relieve the obstruction, and restore enteral nutrition. These factors are expected to allow LGJ to overcome the difficulties in implementing follow-up treatment in patients with gastric cancer with obstruction due to long-term nutrient intake insufficiency, high decomposition, and low synthetic nutrition (28, 33).

Oral feeding can supplement energy and nitrogen sources, reduce negative nitrogen balance, and provide basic conditions for promoting patient recovery. In addition, the latest general rules of tumor nutrition therapy indicate that nutritional therapy not only supplements missing nutrients, but also enhances the body’s immune function and reduces inflammation (34). Rerouting food and digestive juices from the digestive tract restores enteral nutrition and reduces inflammation. Published studies have shown that the systemic inflammatory response promotes tumor growth; therefore, when restoring nutritional choices in patients with obstruction, stimulation of tumor sites should be prevented as much as possible to reduce tumor activation and proliferation induced by the postoperative inflammatory response. In this study, the proportion of patients with PLR<162 in the LGJ group was higher than that in the ES group, which may be because LGJ bypasses the obstruction site after remodeling the digestive tract. This would avoid the continuous stimulation of food or stents to the obstruction site, alleviate the local inflammatory response, and provide personalized enteral nutrition to restore the gastrointestinal immune system.

Our survival analysis showed that LGJ was more beneficial than ES for the survival of patients with advanced gastric cancer complicated by pyloric obstruction. Previous studies have confirmed that gastrojejunostomy can improve chemotherapy compliance and improve the nutritional and metabolic status of such patients (6, 24), The main reason for the survival benefit of this treatment mode is that patients tolerate more chemotherapy cycles, and our study found a significant increase in the number of chemotherapy cycles in patients who underwent NACT after LGJ (6 vs. 4, P<0.001). Notably, individualized enteral nutritional support and adequate nitrogen intake after surgery benefit treatment follow-up.

This study has the following limitations that should be noted when interpreting our results. First, this was a retrospective study; therefore, it may have been subject to selection bias. Second, this study focused on the effect of obstruction relief plus NACT on short-term survival; however, a longer survival evaluation, such as 5-year survival, has yet to be performed. Finally, this study had a small sample size, and further joint multi-center evaluations with larger and more diverse patient cohorts and extended follow-up durations are needed to determine the generalizability and long-term value of our findings.

In patients with locally advanced gastric cancer with pyloric obstruction, the administration of NACT after LGJ can restore their nutritional inflammatory state and allow them to undergo longer chemotherapy cycles. NACT after LGJ also has advantages in potentially reducing the tumor stage and improving patient prognosis. The long-term efficacy of this treatment model requires further evaluation in multi-center, large-sample studies.

## Data Availability

The raw data supporting the conclusions of this article will be made available by the authors, without undue reservation.
